# Adverse childhood and school experiences: a retrospective cross-sectional study examining their associations with health-related behaviours and mental health

**DOI:** 10.1186/s12889-025-21788-3

**Published:** 2025-02-18

**Authors:** Karen Hughes, Mark A Bellis, Kat Ford, Catherine A Sharp, Joanne Hopkins, Rebecca Hill, Katie Cresswell

**Affiliations:** 1 Policy and International Health, World Health Organization Collaborating Centre on Investment for Health and Well-being, Public Health Wales, Cardiff, CF10 4BZ UK; 2 School of Health Sciences, College of Medicine and Health, Bangor University, Wrexham, LL13 7YP UK; 3 Faculty of Health, Innovation, Technology and Science, Liverpool John Moores University, Liverpool, L2 2ER UK

**Keywords:** Adverse childhood experiences, Bullying, School, Mental health, Violence, Smoking, Alcohol use

## Abstract

**Background:**

Adverse childhood experiences (ACEs) increase individuals’ risks of poor health across the life course. For children that suffer household-based ACEs, experiences in other settings such as schools have the potential to exacerbate or mitigate risks of poor health. However, few studies have examined such effects. This study aimed to examine relationships between household-based ACEs, school experiences and adult health outcomes.

**Methods:**

A national cross-sectional household survey (*N* = 1,868 aged 18+) was undertaken in Wales using random quota sampling (November 2022 to March 2023). Measures included nine household-based ACEs; two measures of childhood school experience (having been bullied, sense of school belonging); and adult health outcomes (smoking, binge drinking, low mental wellbeing, mental illness, violence). Associations between ACEs, school experience and health outcomes were examined using chi squared tests and binary logistic regression.

**Results:**

The proportion reporting both having been bullied and lower school belonging increased with ACE count (0 ACEs 6%, 4 + ACEs 51%). Higher ACE count was independently associated with increased risk of all adult health outcomes except binge drinking, while poorer school experience (having been bullied, lower school belonging) was associated with increased risk of low mental wellbeing, mental illness and violence victimisation. For example, adjusted odds of current mental illness rose to 3.98 in those reporting 4 + ACEs (vs. 0 ACEs) and 3.37 in those reporting both having been bullied and lower school belonging (vs. not bullied, higher school belonging). In individuals with 4 + ACEs, adjusted prevalence of current mental illness reduced from 44% in those reporting both having been bullied and lower school belonging to 19% in those reporting not having been bullied and higher school belonging.

**Conclusions:**

For children who grow up in adverse home environments, exposure to further adversity at school may amplify risks of poorer health and life outcomes. However, schools are opportune settings for children who lack safety and support at home to recover from stress, develop resilience and access support. Trauma-informed approaches in schools that recognise the impacts of adversity and support children to overcome it have the potential to improve educational and health outcomes. Further research is needed to identify effective approaches.

**Supplementary Information:**

The online version contains supplementary material available at 10.1186/s12889-025-21788-3.

## Background

Childhood adversity can have lasting impacts on individuals’ health. Over the last few decades, a wealth of research has evidenced and examined how the accumulation of adverse childhood experiences (ACEs) increases individuals’ risks of poorer health across the life course, including through the adoption of health-harming behaviours (e.g. smoking, drug use), involvement in violence, and development of mental and physical ill-health [1–6]. Most studies examining relationships between ACEs and later life outcomes have measured a range of adversities that predominantly affect children in household settings [7, 8]. These include various forms of child maltreatment (e.g. physical abuse, sexual abuse) and challenges affecting household members such as substance misuse, mental illness, domestic violence and incarceration. This range of ACEs can be indicative of a deficiency in the nurturing parental care that children need to thrive. However, children can also face adversity in other settings, such as in schools and communities, and studies show that the risks of exposure to such adversities can be elevated in those who suffer ACEs at home (e.g. involvement in bullying [9, 10]). Consequently, researchers are increasingly incorporating a broader range of adversities into ACE measurement tools to enhance understanding of the extent and impacts of ACEs [8, 11–16].

Various studies have compared expanded ACE measurement tools to traditional tools focused on household ACEs. These have shown that measuring ACEs that occur outside of the household can identify more individuals who have suffered childhood adversity [13], and that some non-household ACEs, such as peer victimisation and witnessing community violence, can be particularly salient at predicting poor behavioural and health outcomes [8, 14]. While some researchers have proposed adapting ACE measurement tools to increase the predictive ability of ACE scales [14, 17], others have focused on the relationships between household ACE exposure and exposure to ACEs in different settings, and to the cumulative effects of exposure to ACEs in different settings. Thus, a study examining youth exposure to individual-level (i.e. household based) and community-level ACEs (e.g. high poverty, high crime) found that while exposure to either ACE type (i.e. individual- or community-level) increased risks of engaging in problem behaviours such as violence and delinquency, experiencing both exacerbated risks [18]. Equally, recent work has focused on (mostly household based) ACEs and peer victimisation in adolescents, finding exposure to either ACEs or peer victimisation to be associated with substance use, with risks being elevated when both are experienced [19]. Similar relationships have been identified between ACEs, bullying and both internalising and externalising behaviours in adolescents [20].

That exposure to adversity in multiple settings increases vulnerability to poorer health and wellbeing is not surprising. Repeated exposure to stress in childhood in the absence of caring adult support can undermine healthy development of the stress response system and brain structures [4, 5, 21], thus children who suffer adversity both at home and elsewhere may have no safe place to recover from stress. Equally, however, positive childhood experiences can improve outcomes for children and may help buffer those who suffer ACEs in the home from developing poor health outcomes [22–24]. Taking a settings approach to ACEs can help develop understanding of how experiences in one setting (e.g. school) might increase or reduce risks of poor health outcomes associated with ACEs in other settings (e.g. at home).

Previous studies in the UK have identified increased risks of poor adult health outcomes in individuals who have suffered household-based ACEs [25] or childhood bullying [26], yet there is a scarcity of knowledge on the links between ACEs and school experiences and their associations with adult health. Here, we use data from a national cross-sectional survey in Wales, UK, to examine relationships between household focused ACE exposure, childhood school experience (having been bullied and sense of school belonging) and adult health outcomes (including alcohol use, smoking, mental ill health and violence).

## Methods

### Data collection

A national cross-sectional household survey was conducted in Wales between November 2022 and March 2023. The target sample size was 2,000 participants aged 18 years and over. A professional market research company was procured to undertake sampling and data collection. A stratified quota sampling methodology was used to obtain a sample representative of population demographics, with Lower Super Output Area (LSOA; small geographical areas with mean population of approximately 1,500) as the sampling unit, stratified by Welsh Health Board and deprivation quintile. In each Health Board (*n* = 7), LSOAs were ranked by their score in the Welsh Index of Multiple Deprivation (WIMD; [22]) and categorised into quintiles. An equal number of LSOAs in each deprivation quintile were then randomly sampled to provide a total sample for each Health Board proportionate to national population share. A total of 200 LSOAs were sampled with a target of 10 interviews per LSOA. Quota samples by age and sex were set for each LSOA, with the inclusion criteria being aged 18 years or over, resident in a sampled LSOA and cognitively able to participate in the survey.

Residential addresses within each sampled LSOA were obtained from the postcode address file. Trained interviewers visited households in sampled LSOAs to invite residents to participate in the study, with participation limited to one individual per household. On contact, household members were shown a letter of authority from Public Health Wales NHS Trust and a participant information sheet explaining the purpose and content of the survey, its voluntary and confidential nature and how findings would be used. All study materials were provided in both Welsh and English language and participants could complete the survey in their language of choice. Informed consent was recorded as part of the survey script. Interviews were conducted face-to-face at participants’ doors by interviewers using Computer Assisted Personal Interviewing. Sensitive questions (including those measuring ACEs and bullying) were self-completed using Computer Assisted Self Interviewing. Following completion of the questionnaire, interviewers provided participants with a thank you letter and information on relevant support services. Just under half (49%) of households contacted and invited to participate agreed to do so, with a total of 2,007 participants completing a questionnaire.

### Study questionnaire

The questionnaire was developed by the research team and used existing, validated measures where possible. Questions measured participants’ demographics, adult health and wellbeing, and a range of questions about childhood experiences (before the age of 18), including exposure to ACEs, bullying at school and sense of school belonging. The questions and response options used in this study are shown in Additional file Table A1. The questionnaire was piloted by the market research company for length, flow and understanding prior to full implementation. The questionnaire took an average of 22 min to complete.

The Centers for Disease Control and Prevention short ACE tool [27] was used to measure exposure to nine ACEs before the age of 18 years: physical, verbal and sexual abuse; parental separation; exposure to domestic violence; and living with a household member with mental illness, alcohol misuse, drug misuse or who had been incarcerated. This widely used tool has shown acceptable psychometric properties in various populations [28, 29]. To reduce participant burden, and in line with previous UK ACE studies [30], we adapted the tool to use a single question to measure exposure to any childhood sexual abuse (“Did an adult or someone at least five years older than you sexually abuse you by touching you or making you undertake any sexual activity with them?”) in place of the three questions used to measure different types of childhood sexual abuse in the original tool (having been touched sexually, made to touch someone else sexually, or forced to have sex by someone at least five years older).

Exposure to bullying was measured by asking participants how often they were bullied by classmates or other children at school (before the age of 18), with responses of ‘sometimes’ or ‘often’ (vs ‘never’ or ‘rarely’) categorised to having been bullied (adapted from [13]). School belonging was measured by a question asking participants whether the statement ‘I felt I belonged in my school’ described them (before the age of 18), with responses dichotomised to higher belonging (responses ‘a lot’ or ‘quite a bit’) and lower belonging (responses ‘somewhat’, ‘a little’ and ‘not at all’).

Health outcomes included current smoking (smoking tobacco daily or occasionally); current binge drinking (reporting drinking five or more alcohol drinks in one day at least once a week); current and lifetime mental illness (receiving treatment for depression, anxiety or another mental illness); and being a victim and perpetrator of violence (having been physically hit or having physically hit someone else in the last 12 months). Low mental wellbeing was measured using the validated Short Warwick-Edinburgh Mental Wellbeing Scale (SWEMWBS; [31] see Additional file Table A1), which has shown robust psychometric properties [32]. Following SWEMWBS guidelines, scores from the seven item scale were summed and transformed from raw to metric scores, with scores more than one standard deviation below the mean categorised as low mental wellbeing (metric scores < 18.16).

Demographics included participants’ sex, age band, ethnicity (self-defined using UK census categories) and postcode. Age was collected using 5-year age bands and categorised into four age groups (18–29; 30–49; 50–69; 70 + years) for analyses. Due to small numbers of participants reporting other than white ethnicities, ethnicity was categorised into two groups: white (including white minorities) and other than white [33]. Postcode was converted to its corresponding LSOA and categorised to a national deprivation quintile (1 = most deprived to 5 = least deprived) using the 2019 WIMD.

For the purpose of analyses, we excluded respondents who could not be assigned to an ACE count due to missing data (*n* = 126), did not answer questions on school bullying and/or school belonging (*n* = 12) or did not provide their age (*n* = 1), leaving a final sample of 1,868 for analysis.

### Statistical analysis

Statistical analyses were conducted using SPSS v24. Consistent with previous ACE studies, positive responses to ACE questions were summed to create a four category ACE count variable for use in analyses (0 ACEs, 1 ACE, 2–3 ACEs, 4 + ACEs). Chi square tests were used initially to examine bivariate relationships between demographics, ACE count, school bullying and school belonging. For analyses of relationships with health outcomes, school bullying (yes, no) and school belonging (higher, lower) were then combined into a four category ‘school experience’ variable. Associations between ACEs, school experience and health outcomes were examined initially using chi squared tests, with independent relationships explored using generalized linear models (GLM; binary logistic) controlling for participant sex, age and deprivation quintile. For outcomes showing strong associations (*p* < 0.001) with both ACE count and school experience, GLM models were also used to generate estimated marginal means.

## Results

Over half (54.4%) of participants were female, 52.9% were aged 50 years or over and 95.7% were of white ethnicity, with a relatively even split across deprivation quintiles (Table 1). A comparison of sample and national demographics is shown in Table A2 (Additional file 1). Overall, 43.5% of participants reported at least one ACE (18.0% 1 ACE, 14.6% 2–3 ACEs, 10.9% 4 + ACEs). The proportion reporting 4 + ACEs reduced with age and increased with deprivation, whilst females were more likely to report multiple ACEs than males. There were no differences in ACE exposure by ethnicity. A quarter (25.3%) of participants reported having been bullied in school and a third (32.7%) reported lower school belonging, with 15.5% reporting both having been bullied and lower belonging (Table 1). School experience was associated with age, with the proportion reporting both having been bullied and lower school belonging being greatest in the youngest age group and reducing with age. There were no significant associations between school experience and other demographics. School experience was strongly associated with ACE count, with 50.7% of those with 4 + ACEs also reporting both having been bullied and lower school belonging, compared with just 6.3% of those with no ACEs (Additional file Figure A1).

The proportion of participants reporting each health outcome ranged from 2.3% for past-year violence perpetration to 27.0% for lifetime mental illness (Table 2). In bivariate analyses, both ACEs and school experience were associated with all outcomes except binge drinking. For example, the proportion of participants reporting lifetime mental illness increased from 15.7% in those reporting no ACEs to 61.8% in those reporting 4 + ACEs, whilst the proportion reporting current low mental wellbeing increased from 8.2% of those who were not bullied with higher school belonging to 36.9% of those reporting both having been bullied and lower school belonging. All health outcomes showed associations with age and all but past-year violence victimisation showed associations with gender and deprivation. Thus, the proportion reporting poorer health outcomes tended to reduce with age and increase with deprivation level (with the exception of binge drinking, which was most commonly reported by the middle deprivation group). Smoking, binge drinking and violence perpetration were more commonly reported by males while low mental wellbeing, and lifetime and current mental illness were more commonly reported by females. There were no significant associations between health outcomes and ethnicity, thus this was not included in multivariate analyses.

Multivariate models were run for each health outcome to examine independent associations with ACE count and school experience, controlling for gender, age and deprivation. Data for binge drinking are provided in Additional File Table A3. Smoking showed strong cumulative associations with ACE count, but no independent relationship with school experience (Table 3). All mental health and wellbeing outcomes showed associations with both ACE count and school experience. For example, compared with individuals with no ACEs, adjusted odds of lifetime mental illness rose to 1.96 in those with 1 ACE, 2.65 in those with 2–3 ACEs and 4.58 in those with 4 + ACEs. Compared with those who were not bullied and reported higher school belonging, odds of lifetime mental illness rose to 1.57 for those who were not bullied but reported lower school belonging, 1.80 for those who were bullied but reported higher school belonging, and 3.55 for those who reported both having been bullied and lower school belonging. Being a victim of violence was associated with both ACE count and school experience, with increased odds significant for those with 4 + ACEs and those who were bullied but reported higher school belonging. Violence perpetration also showed associations with ACE count, with increased odds reaching significance with 4 + ACEs. Odds of violence perpetration were also increased in those reporting having been bullied and lower school belonging, compared with those who were not bullied and had higher school belonging. However, with low numbers reporting violence (victimisation and perpetration), these findings should be treated with caution.

**Table 1:** Sample demographics, ACE count and school experience

		All		ACE count				School experience			
		*n*	%	0 ACEs	1 ACE	2–3 ACEs	4 + ACEs	Not bullied, higher belonging	Not bullied, lower belonging	Bullied, higher belonging	Bullied, lower belonging
**All**	*n*	*1868*	*100.0*	*1056*	*336*	*273*	*203*	*1073*	*322*	*183*	*290*
	%			56.5	18.0	14.6	10.9	57.4	17.2	9.8	15.5
**Sex**	Male	*851*	45.6	58.0	19.6	12.7	9.6	56.3	18.4	10.6	14.7
	Female	*1017*	54.4	55.3	16.6	16.2	11.9	58.4	16.2	9.1	16.2
	*X* ^*2*^						9.105				3.365
	P						**0.028**				0.339
**Age**	18–29	*270*	14.5	41.9	21.5	19.3	17.4	45.6	16.7	10.4	27.4
**group**	30–49	*609*	32.6	52.2	18.4	15.6	13.8	53.5	19.7	9.7	17.1
**(years)**	50–69	*563*	30.1	56.8	19.0	13.9	10.3	58.1	17.2	10.1	14.6
	70+	*426*	22.8	71.6	13.8	11.3	3.3	69.7	14.1	9.2	7.0
	*X* ^*2*^						81.672				70.058
	P						**< 0.001**				**< 0.001**
**Ethnicity**	White	*1788*	95.7	56.5	17.9	14.7	10.9	57.6	17.2	10.1	15.2
	Other than white	*80*	4.3	56.3	20.0	13.8	10.0	53.8	18.8	3.8	23.8
	*X* ^*2*^						0.290				7.071
	P						0.962				0.070
**Deprivation**	(Most) 1	*358*	19.2	53.9	14.2	14.0	17.9	52.8	21.2	10.9	15.1
**quintile**	2	*366*	19.6	58.2	17.5	13.9	10.4	57.1	16.1	9.3	17.5
	3	*382*	20.4	53.7	21.2	15.2	9.9	57.9	15.2	10.5	16.5
	4	*377*	20.2	53.8	19.1	17.8	9.3	53.8	18.3	10.9	17.0
	(Least) 5	*385*	20.6	62.9	17.7	12.2	7.3	65.2	15.6	7.5	11.7
	*X* ^*2*^						36.104				20.130
	P						**< 0.001**				0.065

ACE, adverse childhood experience. Significant P values are shown in bold text

**Table 2:** Health outcomes by demographics, ACE count and school experience

	Current smoker	Current binge drinker	Low mental wellbeing	Lifetime mental illness*	Current mental illness*	Violence victim	Violence perpetrator
**All**							
*n*	*239*	*454*	*304*	*496*	*261*	*68*	*43*
%	12.8	24.3	16.3	27.0	14.2	3.6	2.3
**Sex**							
Male	14.7	32.1	13.4	20.4	9.8	4.0	3.2
Female	11.2	17.8	18.7	32.6	17.9	3.3	1.6
*X* ^*2*^	5.026	51.372	9.502	34.328	24.913	0.562	5.271
P	**0.025**	**< 0.001**	**0.002**	**< 0.001**	**< 0.001**	0.454	**0.022**
**Age group**							
18–29	17.4	34.8	22.6	30.9	18.1	13.3	11.1
30–49	14.1	31.4	14.4	30.3	16.7	3.6	1.6
50–69	14.7	24.0	17.4	30.3	16.0	1.4	0.4
70+	5.4	8.0	13.4	15.6	5.9	0.5	0.2
*X* ^*2*^	28.907	94.435	12.547	36.267	31.716	92.437	111.927
P	**< 0.001**	**< 0.001**	**0.006**	**< 0.001**	**< 0.001**	**< 0.001**	**< 0.001**
**Ethnicity**							
White	12.8	24.6	15.9	27.2	14.3	3.6	2.2
Other than white	12.5	17.5	23.8	23.4	11.7	5.0	5.0
*X* ^*2*^	0.006	2.103	3.428	0.535	0.419	0.441	2.705
P	0.936	0.147	0.064	0.464	0.518	0.507	0.100
**Deprivation quintile**							
(Most) 1	17.0	19.0	24.6	30.9	19.8	4.7	4.7
2	15.8	22.7	19.7	28.3	16.3	1.9	1.9
3	14.4	29.1	14.4	23.9	11.9	4.5	1.8
4	9.3	24.9	14.9	30.1	14.2	4.2	2.1
(Least) 5	7.8	25.5	8.6	22.2	9.3	2.9	1.0
*X* ^*2*^	22.516	11.062	39.541	11.116	19.402	6.148	12.935
P	**< 0.001**	**0.026**	**< 0.001**	**0.025**	**< 0.001**	0.188	**0.012**
**ACE count**							
0	7.4	23.1	9.7	15.7	8.0	1.4	0.9
1	14.0	25.0	14.6	29.0	13.7	3.6	2.4
2–3	18.3	24.2	23.4	41.8	18.3	5.1	2.9
4+	31.5	29.6	43.8	61.8	41.7	13.3	8.9
*X* ^*2*^	99.413	3.959	158.145	219.546	160.284	70.568	49.261
P	**< 0.001**	0.266	**< 0.001**	**< 0.001**	**< 0.001**	**< 0.001**	**< 0.001**
**School experience**							
Not bullied, higher belonging	9.2	22.8	8.2	17.1	7.8	1.6	0.9
Not bullied, lower belonging	16.8	25.8	22.4	29.9	15.0	3.4	2.2
Bullied, higher belonging	13.1	27.3	20.2	33.5	19.0	7.7	2.7
Bullied, lower belonging	21.4	26.2	36.9	56.7	34.2	9.0	7.2
*X* ^*2*^	35.977	3.118	152.679	185.218	131.589	44.810	40.590
P	**< 0.001**	0.374	**< 0.001**	**< 0.001**	**< 0.001**	**< 0.001**	**< 0.001**

*31 participants reported ‘prefer not to say’, thus N for outcome = 1837. ACE, adverse childhood experience. Significant P values are shown in bold text

**Table 3:** Adjusted odds ratios (AORs) for health outcomes by demographics, ACE count and school experience

	Current smoker		Low mental wellbeing		Lifetime mental illness*		Current mental illness*		Violence victim		Violence perpetrator	
	AOR (95% CI)	P	AOR (95% CI)	P	AOR (95% CI)	P	AOR (95% CI)	P	AOR (95% CI)	P	AOR (95% CI)	P
**Sex**												
Female	0.66 (0.49–0.88)	**0.004**	1.54 (1.17–2.02)	**0.002**	1.95 (1.54–2.47)	**< 0.001**	2.03 (1.51–2.74)	**< 0.001**	0.77 (0.46–1.29)	0.324	0.39 (0.19–0.77)	**0.007**
**Age group (years)**												
18–29	2.39 (1.38–4.13)	**0.002**	0.95 (0.61–1.49)	0.907	1.33 (0.88–2.01)	0.176	1.90 (1.10–3.29)	**0.022**	20.26 (4.74–86.58)	**< 0.001**	31.46 (4.17–237.50)	**0.001**
30–49	2.14 (1.31–3.51)	**0.002**	0.62 (0.42–0.93)	**0.019**	1.58 (1.12–2.22)	**0.009**	2.08 (1.29–3.37)	**0.003**	5.63 (1.30-24.44)	**0.021**	4.82 (0.60-38.56)	0.139
50–69	2.55 (1.56–4.17)	**< 0.001**	0.99 (0.68–1.45)	0.961	1.86 (1.32–2.62)	**< 0.001**	2.35 (1.45–3.81)	**0.001**	2.20 (0.46–10.56)	0.324	1.14 (0.10-12.75)	0.917
70+	Ref	**0.002**	Ref	**0.027**	Ref	**0.003**	Ref	**0.006**	Ref	**< 0.001**	Ref	**< 0.001**
**Deprivation quintile**												
(Most) 1	2.02 (1.25–3.27)	**0.004**	3.00 (1.90–4.75)	**< 0.001**	1.26 (0.88–1.83)	0.211	1.98 (1.24–3.16)	**0.004**	1.05 (0.45–2.42)	0.912	3.81 (1.16–12.44)	**0.027**
2	2.09 (1.29–3.39)	**0.003**	2.52 (1.58–4.02)	**< 0.001**	1.21 (0.84–1.75)	0.302	1.71 (1.06–2.75)	**0.027**	0.43 (0.16–1.17)	0.100	1.39 (0.37–5.16)	0.623
3	1.80 (1.11–2.92)	**0.017**	1.60 (0.99–2.59)	0.057	0.90 (0.62–1.31)	0.589	1.09 (0.66–1.80)	0.733	1.28 (0.57–2.90)	0.554	1.61 (0.44–5.93)	0.473
4	1.07 (0.63–1.80)	0.803	1.64 (1.02–2.66)	**0.041**	1.31 (0.92–1.88)	0.137	1.44 (0.89–2.33)	0.137	1.21 (0.53–2.77)	0.656	1.91 (0.53–6.85)	0.321
(Least) 5	Ref	**0.002**	Ref	**< 0.001**	Ref	0.186	Ref	**0.016**	Ref	0.208	Ref	0.099
**ACE count**												
0	Ref	**< 0.001**	Ref	**< 0.001**	Ref	**< 0.001**	Ref	**< 0.001**	Ref	**< 0.001**	Ref	**0.035**
1	1.86 (1.25–2.76)	**0.002**	1.44 (0.98–2.11)	0.062	1.96 (1.45–2.66)	**< 0.001**	1.60 (1.08–2.39)	**0.020**	1.85 (0.84–4.12)	0.129	1.93 (0.70–5.34)	0.204
2–3	2.55 (1.69–3.85)	**< 0.001**	1.89 (1.30–2.75)	**0.001**	2.65 (1.93–3.63)	**< 0.001**	1.67 (1.10–2.51)	**0.015**	2.20 (0.99–4.89)	0.052	1.80 (0.62–5.21)	0.281
4+	4.58 (2.96–7.09)	**< 0.001**	3.73 (2.50–5.56)	**< 0.001**	4.58 (3.16–6.66)	**< 0.001**	3.98 (2.62–6.03)	**< 0.001**	5.19 (2.40-11.23)	**< 0.001**	4.35 (1.58–11.96)	**0.004**
**School experience**												
Not bullied, higher belonging	Ref	0.310	Ref	**< 0.001**	Ref	**< 0.001**	Ref	**< 0.001**	Ref	**0.044**	Ref	0.179
Not bullied, lower belonging	1.37 (0.93–2.01)	0.106	2.63 (1.83–3.78)	**< 0.001**	1.57 (1.15–2.14)	**0.004**	1.58 (1.05–2.37)	**0.027**	1.37 (0.61–3.08)	0.446	1.48 (0.52–4.22)	0.462
Bullied, higher belonging	0.92 (0.56–1.54)	0.761	2.08 (1.33–3.27)	**0.001**	1.80 (1.24–2.63)	**0.002**	2.04 (1.28–3.25)	**0.003**	3.04 (1.37–6.75)	**0.006**	1.44 (0.42–4.88)	0.558
Bullied, lower belonging	1.23 (0.81–1.85)	0.334	3.99 (2.74–5.81)	**< 0.001**	3.55 (2.56–4.91)	**< 0.001**	3.37 (2.28–4.98)	**< 0.001**	2.02 (0.96–4.25)	0.064	2.73 (1.07–6.97)	**0.036**

*31 participants reported ‘prefer not to say’, thus N for outcome = 1837. CI, confidence interval; ACE, adverse childhood experience; Ref, reference category. Reference category for Sex is male. P values in Ref rows relate to the overall contribution made by each independent variable to the model. Significant P values are shown in bold text

**Fig. 1:**
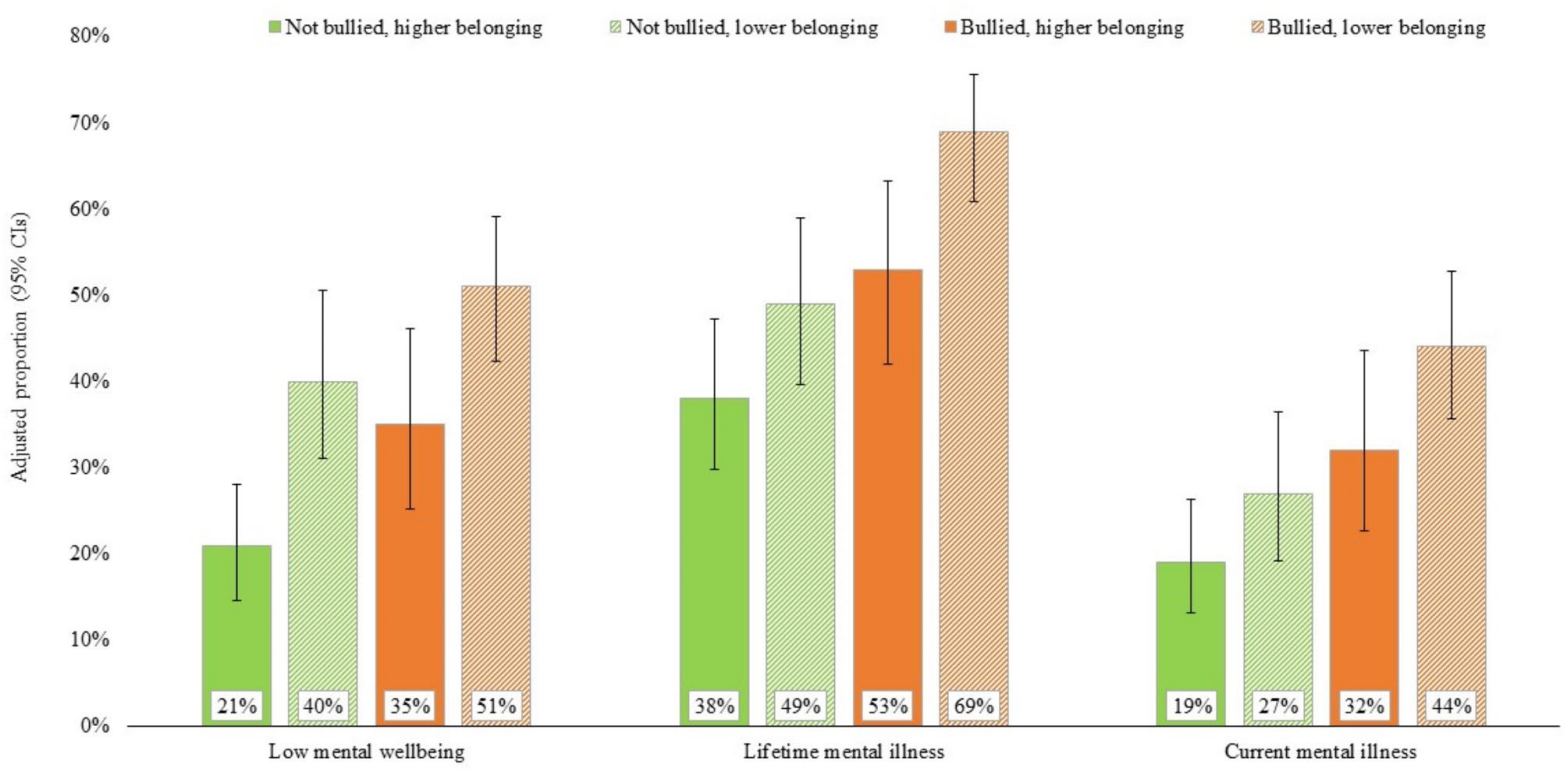
Adjusted prevalence of mental health and wellbeing outcomes for individuals with 4 + ACEs by school experience Footnote: Adjusted proportions are estimated marginal means for individuals with 4 + ACEs. Models controlled for sex, age and deprivation quintile. ACE, adverse childhood experience; CIs, confidence intervals

GLM models were used to estimate demographically adjusted proportions (estimated marginal means) with low mental wellbeing, lifetime mental illness and current mental illness by ACE count and school experience (Fig. 1; Additional file Table A4). For individuals with 4 + ACEs, adjusted prevalence of low mental wellbeing more than halved from 51% in those who were bullied and had low school belonging to 21% in those who were not bullied and had higher school belonging. Equivalent reductions were from 69 to 38% for lifetime mental illness and from 44 to 19% for current mental illness.

## Discussion

Safe and supportive childhood environments are integral to providing children with the best opportunities for health throughout life. The harms that ACEs impose on health across the life course and the need to support children affected by ACEs are increasingly well recognised. Whilst studies on ACEs have predominantly focused on household-based adversities, there is growing interest in how childhood adversities arising in other settings may interact with household ACEs, potentially resulting in cumulative risks to health [18, 19]. Moreover, other settings also offer opportunities to mitigate against harms associated with household ACEs, including through building resilience in those who may be affected [34, 35]. Here, focusing on school settings and using a national sample of adults in Wales, we found that household ACEs and school experiences were independently associated with adult health (Table 3). Thus, consistent with other studies [2, 36, 37], ACEs showed strong relationships with smoking, low mental wellbeing, and both current and lifetime mental illness. Having been bullied and experiencing lower school belonging was also independently increased the likelihood of all three mental health outcomes. Findings also suggest that higher exposure to ACEs at home and both being bullied and lower sense of school belonging are more likely to co-occur in the same individuals. Thus, only 6% of individuals with no ACEs reported both being bullied and lower school belonging compared to 51% of those with four or more ACEs.

School systems need to be aware of the impacts ACEs can have on their pupils, including their relationships with poor belonging in school and vulnerability to other traumas such as bullying. Children exposed to ACEs at home may enter the school system with poorer foundational skills (e.g. language, numeracy, literacy), social skills and executive functioning (e.g. working memory, attention) [38–40]; affecting their ability to concentrate and learn in classroom environments, bond with peers and trust teachers and other professionals attempting to support their development. Children may internalise the impacts of home-based trauma and disengage from their educational environment, with ACEs having been associated with lower school engagement, greater school absenteeism, dropout and ultimately lower grade and qualification attainment [41–46]. They may also externalise the consequences of ACEs, increasing disruptive and anti-social behaviour along with risk of suspension and exclusionary discipline [39, 47]. Whilst our study measured bullying victimisation, studies elsewhere show children with ACEs can be at increased risk of perpetrating bullying [9, 10]. Results here suggest those with four or more ACEs (vs. no ACEs) were more than four time more likely to be involved in violence (in the last year) even as adults; a pattern of behaviour that typically begins in childhood [48, 49].

Schools already play an important role in developing children’s social and emotional skills and their importance as a safe and nurturing setting for children who suffer ACEs is increasingly being recognised [50, 51]. Alongside education, schools can offer children a wealth of benefits including safety, friendship, trusted adult support, life skills and access to specialist services. Positive school experiences can support the development of resilience in children who suffer ACEs and may help improve their educational outcomes [52] and support better health in later life. In our study, adjusted prevalence of low mental wellbeing and mental illness was substantially reduced when adults reported more positive school experiences. For example, the adjusted proportion of current mental illness more than halved from 44% in those who were bullied and had lower school belonging to 19% in those who were not bullied and had higher school belonging (Table 3). Thus, our study contributes to knowledge on the vulnerability of children who suffer ACEs to poorer health and the potential of positive school experiences in reducing this vulnerability. Findings can inform the development of policy and practice within schools to support children affected by ACEs. In Wales, for example, such work has included the delivery of training to staff across primary, secondary and tertiary education levels to equip teachers and other school staff with the tools to identify and address the impact of ACEs. Work has also examined how schools support children with ACEs and how this support can be strengthened, including the importance of partnership working between schools and other agencies who engage with children and families for continuation of support [53, 54].

More research is needed to examine school-related protective factors for children affected by ACEs, which may include features of child-teacher relationships, school culture, school-based activities and specific interventions that support children who suffer adversity [41, 44]. A growing body of literature is identifying evidence for the effectiveness of school-based interventions for children affected by trauma [55]. Whilst many such interventions are delivered by specialists such as therapists and psychologists, and therefore may be cost-prohibitive to adopt universally across educational systems, evidence is also beginning to emerge for interventions that can be delivered by non-clinicians [56]. At a whole-school level, programmes for trauma-informed schools have been implemented in several countries to increase understanding of ACEs and other sources of trauma amongst all staff and provide the tools and settings necessary for children to de-stress, build resilience and better integrate into school environments [57, 58]. Some studies have reported benefits to school performance, child behaviour and child-teacher relationships linked to the implementation of trauma-informed approaches in schools [59–61]. However, the evidence base for the effectiveness of trauma-informed schools in improving outcomes for children remains scant and further, more rigorous research is needed to inform practice in this important area [61, 62].

Our results, like others, suggest experiencing ACEs alone and in combination with school-based trauma such as bullying is far from rare. Here, nearly half of all respondents reported at least one ACE as a child and more than one in ten experienced more than four ACEs, while more than a third reported being bullied at school. The costs associated with such trauma continuing to affect further generations is likely to be substantive with estimates that ACEs in the UK result in costs of £78.6 billion per year (2.8% of GDP) [63]. A fraction of this redirected at preventing ACEs in home environments through better supported parenting and creating trauma-informed schools is likely to provide competitive returns on investment compared to other calls on public monies.

Our study had several limitations that affect the generalisability of findings. While compliance with the survey (49%) was similar to that seen in previous general population ACE surveys [25], findings may be affected by selection bias, and we are unable to identify how the childhood experiences and health outcomes of those choosing not to participate may have affected results. Equally, 6.8% (*n* = 138) of all those who participated in the survey could not be included in analyses as they chose not to answer questions on certain ACEs or school experiences. This may have resulted in under-representation of those who experienced ACEs or poorer school experiences. Exposure to ACEs and school experience were measured retrospectively and as such are subject to recall bias and willingness to report. However, we used a widely used tool to measure ACEs, and the prevalence of ACEs reported by our sample was equivalent to that measured in previous general population studies in the UK [25]. We adapted the ACE tool to merge three questions on different forms of childhood sexual abuse into a single question covering all three forms, yet this may have impacted reported levels of sexual abuse. Our measurement of school experience was restricted to single measures of bullying victimisation and sense of school belonging, with a combined variable used to examine exposure to one or both experiences. While we based our measure of bullying exposure on a question included in a previous retrospective ACE survey [13] (adapted to specify school as the setting and maintain consistency in survey response options; see Additional file Table A1), we did not provide participants with a definition of bullying and reports were therefore subject to participants’ personal perceptions of what this concept means [64]. Future research would benefit from examination of a greater range and depth of school experiences, including through mixed method or qualitative research approaches that provide greater perspective and context to inform preventive action.

Outcome measurements were self-reported and thus subject to bias in reporting. Our measurement of mental illness focused on receipt of treatment and may have missed individuals who experienced mental illness but did not seek or were unable to access treatment. Studies show demographic differences in help seeking for mental illness with, for example, males being less likely to seek help than females [65]; this effect may have affected findings (i.e. greater odds of reporting mental illness by females). Further, the numbers reporting past year violence victimisation and perpetration were low, restricting analytical power. While ACEs, and to a lesser extent school experience, showed associations with violence, these relationships require examining in a larger sample. Analysis of ethnicity was also restricted by low sample, with just 4.6% reporting other than white ethnicity (vs. 6.2% in the Welsh general population). While there was no significant difference in ACE count by ethnicity, studies elsewhere with more ethnically heterogeneous samples have shown higher ACE prevalence among non-White ethnic minority groups (e.g. USA [66]). Previous UK studies have also identified variation in ACE exposure by ethnicity, with reported ACE counts in an English study being lower in those of Asian ethnic backgrounds and higher in other ethnic minority groups [67]. Ethnicity may also influence school experiences [68, 69]. Ethnic minority populations may experience racial discrimination in schools, increasing their vulnerability to bullying and lower school belonging [70]. Thus, further work is needed to examine relationships between ACEs, school experiences and health in individuals from ethnic minority backgrounds. Finally, as our study was cross-sectional, findings do not necessarily indicate causal relationships between ACEs, school experience and outcomes measured.

## Conclusions

Children who suffer ACEs are at increased risk of poor educational outcomes, and of developing poorer health and social wellbeing across the life course. For those who suffer ACEs at home, exposure to further adversity at school is likely to amplify this risk. However, schools are also opportune settings for children who lack safety and support at home to recover from stress and build resilience. Such children may require a disproportionate amount of early support in order to properly engage them in educational systems. Ensuring appropriate support is available requires schools to adopt trauma-informed approaches where staff understand the impact of ACEs and feel equipped to provide appropriate support. However, positive school experiences are also likely to confer substantial benefits to school attendance, staff satisfaction and educational outcomes and consequently to broader society.

## Electronic supplementary material

Below is the link to the electronic supplementary material.

Supplementary Material 1

## Data Availability

The dataset analysed in the current study is available from the corresponding author on reasonable request.

## Abbreviations

ACE: Adverse childhood experience
AOR: Adjusted odds ratio
CI: Confidence interval
GLM: Generalized linear model
LSOA: Lower Super Output Area
Ref: Reference category
SWEMWBS: Short Warwick-Edinburgh Mental Wellbeing Scale
WIMD: Welsh Index of Multiple Deprivation
