# Supplementary material for: Adverse childhood and school experiences: a retrospective cross-sectional study examining their associations with health-related behaviours and mental health
Source: BMC Public Health. 2025 Feb 18;25:672. doi: 10.1186/s12889-025-21788-3 (PMC11837321; doi:10.1186/s12889-025-21788-3)
Supplement: Supplementary file 1 — Supplementary Material 1 [file 12889_2025_21788_MOESM1_ESM.docx]

**Table A1. Questions and qualifying responses for independent variables**

|  | **Question *(response options)*** | **Qualifying response** | |
| --- | --- | --- | --- |
| **ACEs** | All ACE questions were preceded by the statement “While you were growing up, before the age of 18 years...” | |  |
| *Physical abuse* | How often did a parent or adult in your home ever hit, beat, kick, or physically hurt you in any way? This does not include gentle smacking for punishment. *(never; once; more than once; prefer not to say)* | Once or more than once | |
| *Verbal abuse* | How often did a parent or adult in your home ever swear at you, insult you, or put you down? *(never; once; more than once; prefer not to say)* | More than once | |
| *Sexual abuse* | Did an adult or someone at least five years older than you sexually abuse you by touching you or making you undertake any sexual activity with them? *(yes; no; prefer not to say)* | Yes | |
| *Parental separation* | Were your parents ever separated or divorced? *(yes; no; prefer not to say)* | Yes | |
| *Domestic violence* | How often did your parents or adults in your home ever slap, kick, punch, or beat each other up? *(never; once; more than once; prefer not to say)* | Once or more than once | |
| *Mental illness* | Did you live with anyone who was depressed, mentally ill or suicidal? *(yes; no; prefer not to say)* | Yes | |
| *Alcohol abuse* | Did you live with anyone who was a problem drinker or alcoholic? *(yes; no; prefer not to say)* | Yes | |
| *Drug abuse* | Did you live with anyone who used illegal street drugs or abused prescription medications? *(yes; no; prefer not to say)* | Yes | |
| *Incarceration* | Did you live with anyone who served time or was sentenced to serve time in a prison or young offenders' institution? *(yes; no; prefer not to say)* | Yes | |
| **School experience** | Questions were preceded by the statement “While you were growing up, before the age of 18 years...” | | |
| *Bullied* | How often were you bullied by classmates or other children at school? *(Never; rarely; sometimes; often; prefer not to say)* | Sometimes or often | |
| *School belonging* | To what extent would the statements below have described you...? “I felt I belonged in my school” (*not at all; a little; somewhat; quite a bit; a lot; prefer not to say*) |  | |
|  | *Higher belonging* | A lot, quite a bit | |
|  | *Lower belonging* | Somewhat, a little, not at all | |
| **Health outcomes** |  | | |
| *Current smoker* | In terms of smoking tobacco, which of the following describes you? *(I currently smoke tobacco; I used to smoke tobacco, but do not smoke it now; I have never smoked tobacco; prefer not to say)* | I currently smoke tobacco | |
| *Current binge drinker* | Currently, on how many days a week do you drink five or more alcoholic drinks in one day (e.g. glasses of wine, beers, shots of spirits)? (*0; 1; 2; 3; 4; 5; 6; 7; prefer not to say); Only asked to those who reported drinking alcohol, with those reporting never or not currently drinking alcohol coded to ‘no’ for binge drinking.* | 1 or more | |
| *Low mental wellbeing* | *Short Warwick-Edinburgh Mental Wellbeing Scale (SWEMWBS)*  Below are some statements about feelings and thoughts. Please select the answer that best describes your experience of each over the last 2 weeks… *(scoring: none of the time = 1, rarely = 2, some of the time = 3, often = 4, all of the time = 5; prefer not to say)* | Metric scores <18.16  Using SWEMWBS guidelines, scores for individual questions were summed and transformed into metric scores | |
|  | I’ve been feeling optimistic (positive) about the future |  |  |
|  | I’ve been feeling useful |  |  |
|  | I’ve been feeling relaxed |  |  |
|  | I’ve been dealing with problems well |  |  |
|  | I’ve been thinking clearly |  |  |
|  | I’ve been feeling close to other people |  |  |
|  | I’ve been able to make up my own mind about things |  |  |
| *Lifetime mental illness* | Are you currently receiving, or have you ever received, treatment for depression, anxiety or another mental illness? *(yes, currently; yes, in the past; no, never; prefer not to say)* | Yes, currently, yes, in the past | |
| *Current mental illness* | *As above* | Yes, currently | |
| *Violence victim* | In the past 12 months, have you been physically hit by someone else? *(yes; no; prefer not to say)* | Yes | |
| *Violence perpetrator* | In the past 12 months, have you physically hit someone else? *(yes; no; prefer not to say)* | Yes | |

ACE, Adverse childhood experience. SWEMWBS© NHS Health Scotland, University of Warwick and University of Edinburgh, 2008, all rights reserved.

**Table A2: Comparison of sample with Welsh national demographics**

|  | **Sample** | **National population** |
| --- | --- | --- |
| **Sex** |  |  |
| Male | 46% | 49% |
| Female | 54% | 51% |
| **Age group (years)** |  |  |
| 18-29 | 14% | 19% |
| 30-49 | 33% | 29% |
| 50-69 | 30% | 32% |
| 70+ | 23% | 19% |
| **Deprivation quintile*** |  |  |
| (Most) 1 | 19% | 19% |
| 2 | 20% | 20% |
| 3 | 20% | 21% |
| 4 | 20% | 21% |
| (Least) 5 | 21% | 20% |
| **Ethnicity**^ |  |  |
| White | 96% | 95% |
| Other than White | 4% | 5% |

*Welsh Index of Multiple Deprivation 2019 <https://www.gov.wales/welsh-index-multiple-deprivation-full-index-update-ranks-2019>.

^National population ethnicity figures are for all ages as figures are not published for other than white ethnic groups aged 65+ due to small numbers; Stats Wales, <https://statswales.gov.wales/Catalogue/Equality-and-Diversity/Ethnicity>.

**Table A3: Adjusted odds ratios (AORs) for current binge drinking**

|  | **Current binge drinker** | |
| --- | --- | --- |
|  | AOR (95%CI) | P |
| **Sex** |  |  |
| Female | 0.42 (0.34-0.53) | <0.001 |
| **Age group (years)** |  |  |
| 18-29 | 6.66 (4.26-10.41) | <0.001 |
| 30-49 | 6.09 (4.07-9.09) | <0.001 |
| 50-69 | 3.85 (2.56-5.78) | <0.001 |
| 70+ | Ref | <0.001 |
| **Deprivation quintile** |  |  |
| (Most) 1 | 0.59 (0.41-0.85) | 0.005 |
| 2 | 0.75 (0.53-1.07) | 0.114 |
| 3 | 1.10 (0.79-1.54) | 0.575 |
| 4 | 0.91 (0.64-1.28) | 0.591 |
| (Least) 5 | Ref | 0.007 |
| **ACE count** |  |  |
| 0 | Ref | 0.668 |
| 1 | 0.90 (0.67-1.22) | 0.515 |
| 2-3 | 0.96 (0.68-1.35) | 0.805 |
| 4+ | 1.18 (0.80-1.74) | 0.412 |
| **School experience** |  |  |
| Not bullied, higher belonging | Ref | 0.784 |
| Not bullied, lower belonging | 1.02 (0.75-1.39) | 0.912 |
| Bullied, higher belonging | 1.16 (0.79-1.69) | 0.456 |
| Bullied, lower belonging | 0.92 (0.65-1.30) | 0.624 |

AOR, adjusted odds ratio; CI, confidence interval; ACE, adverse childhood experience; Ref, reference category. Reference category for Sex is male. P values in Ref rows relate to the overall contribution made by each independent variable to the model.

**Table A4: Demographically adjusted proportions (estimated marginal means; EMM) with low mental wellbeing, lifetime mental illness and current mental illness by ACE count and school experience**

|  |  | **Low mental wellbeing** | **Lifetime mental illness*** | **Current mental illness*** |
| --- | --- | --- | --- | --- |
|  |  | EMM (95% CI) | EMM (95% CI) | EMM (95% CI) |
| **0 ACEs** | Not bullied, higher belonging | 0.06 (0.05-0.08) | 0.12 (0.10-0.14) | 0.06 (0.04-0.07) |
|  | Not bullied, lower belonging | 0.15 (0.12-0.20) | 0.17 (0.14-0.22) | 0.08 (0.06-0.12) |
|  | Bullied, higher belonging | 0.13 (0.09-0.18) | 0.20 (0.14-0.26) | 0.11 (0.07-0.16) |
|  | Bullied, lower belonging | 0.22 (0.16-0.28) | 0.32 (0.26-0.40) | 0.17 (0.12-0.23) |
| **1 ACE** | Not bullied, higher belonging | 0.09 (0.06-0.13) | 0.21 (0.17-0.26) | 0.09 (0.06-0.12) |
|  | Not bullied, lower belonging | 0.21 (0.15-0.28) | 0.29 (0.23-0.37) | 0.13 (0.09-0.19) |
|  | Bullied, higher belonging | 0.17 (0.11-0.25) | 0.32 (0.24-0.42) | 0.16 (0.11-0.24) |
|  | Bullied, lower belonging | 0.28 (0.21-0.37) | 0.48 (0.40-0.57) | 0.24 (0.17-0.33) |
| **2-3 ACEs** | Not bullied, higher belonging | 0.12 (0.08-0.16) | 0.26 (0.21-0.33) | 0.09 (0.06-0.13) |
|  | Not bullied, lower belonging | 0.26 (0.19-0.33) | 0.36 (0.29-0.44) | 0.13 (0.09-0.19) |
|  | Bullied, higher belonging | 0.21 (0.15-0.30) | 0.39 (0.30-0.49) | 0.17 (0.11-0.25) |
|  | Bullied, lower belonging | 0.34 (0.27-0.43) | 0.56 (0.48-0.64) | 0.25 (0.18-0.33) |
| **4+ ACEs** | Not bullied, higher belonging | 0.21 (0.15-0.28) | 0.38 (0.30-0.47) | 0.19 (0.13-0.26) |
|  | Not bullied, lower belonging | 0.40 (0.31-0.50) | 0.49 (0.40-0.59) | 0.27 (0.19-0.36) |
|  | Bullied, higher belonging | 0.35 (0.25-0.46) | 0.53 (0.42-0.63) | 0.32 (0.23-0.44) |
|  | Bullied, lower belonging | 0.51 (0.42-0.59) | 0.69 (0.61-0.76) | 0.44 (0.36-0.53) |

*31 participants reported prefer not to say, thus N for outcome =1,837. CI, confidence interval; ACE, adverse childhood experience.

**Figure A1: ACE count by school experience**

ACE, Adverse childhood experience.
